# A Risk-Based Framework for Hospital Compounding: Integrating Degradation Mechanisms and Predictive Toxicology

**DOI:** 10.3390/pharmaceutics17091202

**Published:** 2025-09-16

**Authors:** Philippe-Henri Secretan, Maxime Annereau, Bernard Do

**Affiliations:** 1 Matériaux et Santé, Université Paris-Saclay, 91400 Orsay, France; 2 Clinical Pharmacy Department, Gustave Roussy Cancer Campus, 94805 Villejuif, France; 3 Institut des Sciences Moléculaires d’Orsay, Centre National de la Recherche Scientifique CNRS, Université Paris Saclay, 91400 Orsay, France

**Keywords:** hospital compounding, risk-based formulation, degradation profiling, in silico toxicology, critical quality attributes (cQAs), 3D printing in pharmaceutics, personalized medicine, mutagenic impurities

## Abstract

**Background/Objectives**: Hospital compounding is essential for the delivery of patient-tailored therapies—particularly for pediatric and oncology patients and other groups requiring precise dosing. Its role is expected to grow as, for instance, the UK MHRA’s new Guidance on Decentralised Manufacturing promotes alternative manufacturing pathways that integrate hospital preparation units. However, drug substances that remain stable in commercial oral formulations may undergo rapid degradation under alternative conditions (e.g., aqueous suspension, light exposure, or in the presence of specific excipients). Despite these risks, formulation strategies in hospital compounding often rely on empirical practices and lack structured guidance regarding stability, impurity control, and reproducibility. **Methods**: This study proposes a risk-based scientific framework for formulation design, integrating degradation profiling with predictive toxicology. Potential degradation pathways (hydrolytic, oxidative, and photolytic) are systematically identified through forced-degradation studies combined with ab initio modeling. These risks are translated into formulation strategies using a structured decision tree encompassing solvent selection, pH adjustment, excipient compatibility, and packaging considerations, even in the absence of a pharmacopeial monograph. The toxicological relevance of degradation products is evaluated using in silico approaches aligned with ICH M7 guidelines, thereby defining critical quality attributes (cQAs) and critical process parameters (CPPs). **Results**: The applicability of the framework is demonstrated through hospital compounding case studies, with further extension toward advanced applications such as semi-solid extrusion (SSE) 3D printing. **Conclusions**: By integrating mechanistic understanding of drug degradation into formulation planning, the proposed framework enhances the safety, reproducibility, and quality of compounded preparations. This approach reinforces Good Preparation Practices (GPPs) and is consistent with international quality-by-design (QbD) principles in the context of personalized medicine.

## 1. Introduction

The rising demand for personalized medicine has transformed the landscape of pharmaceutical care, particularly in pediatric, oncologic, and rare disease populations, where standardized dosage forms often fail to meet individual patient needs [1,2]. Hospital-based compounding has emerged as a crucial enabler of this transformation by providing dose-adapted, patient-specific formulations when licensed alternatives are unavailable or unsuitable [3].

Despite its clinical value, hospital compounding often lacks robust guidance on stability, impurity risk, and reproducibility [4]. Unlike industrial drug manufacturing, which is regulated under Good Manufacturing Practice (GMP) standards and benefits from extensive stability data and validated monographs, hospital compounding is governed by frameworks such as Good Preparation Practices (GPP) or USP chapters <795> [5], <797> [6], and <800> [7]. These guidelines primarily focus on hygiene, sterility, and safety during preparation, but offer limited support for addressing chemical degradation, excipient compatibility, or formulation strategy in the absence of complete regulatory data.

The limited support for addressing chemical degradation is an important hurdle as, to allow for dose adjustment, the current compounded preparations for oral delivery often consist of suspension and oral solution, in which the active pharmaceutical ingredient is amenable to degrade by hydrolysis, oxidation or photodegradation [8,9,10]. This is also an important obstacle for the development of new promising semi-solid dosage forms compounded by 3D printing, an emerging technique already implemented in some hospital compounding units [11,12,13].

Recent advances in analytical chemistry and computational modeling improve prediction and characterization of degradation pathways using forced-degradation studies, MS^n^ [14,15,16,17], and density functional theory (DFT) [18,19,20,21,22]. These tools are common in industry but underused in compounding, where they could markedly improve safety and quality. Moreover, the lack of pharmacopoeial monographs or standardized impurity limits for many extemporaneously prepared medicines leaves hospital pharmacists with limited resources to assess chemical risk or establish critical quality attributes (cQAs).

We propose a framework that integrates degradation profiling, in silico toxicology, and formulation decision-making aligned with ICH Q8–Q12 [23,24,25,26,27] and M7 [28]. This framework is designed to help hospital pharmacists identify and control degradation risks through a structured process that defines critical process parameters (CPPs), supports safe excipient and container choices, and guides risk-based compounding decisions, even under real-world constraints.

To demonstrate the practical utility of this approach, we apply the framework to a set of representative hospital-compounded drugs, including pediatric and oncology formulations. Where appropriate, we also explore how this degradation-informed strategy may be extended to emerging technologies such as semi-solid extrusion (SSE) 3D printing, which offers flexible, on-demand manufacturing of personalized doses. However, this application is considered exploratory rather than central to the current work.

Our aim is to bridge the scientific gap between degradation knowledge and real-world hospital compounding by providing a pragmatic, risk-based formulation tool that improves safety, traceability, and regulatory alignment in personalized medicine.

## 2. Materials and Methods

### 2.1. Degradation Risk Identification and Profiling by Experimental and Theoretical Approaches

Forced degradation studies were conducted on representative drug substances using ICH Q1A(R2) [29] guidance to identify potential degradation pathways under hydrolytic, oxidative, photolytic, and thermal stress conditions. We employed LC–MS/MS and high-resolution mass spectrometry (HRMS) to characterize major degradation products [15,16,17]. These data were used to establish chemical liability profiles for common classes of small molecules used in hospital settings, such as anticancer agents, antivirals, and anticoagulants.

Empirical data was complemented with ab initio modeling and DFT in order to predict susceptible moieties and potential intermediates. After geometric optimization of the studied active pharmaceutical ingredient, HOMO–LUMO gaps, mapped electrostatic potential, average local ionization energy maps as well as Fukui function [19,20,21,22,30,31,32] have been obtained to support the degradation routes. These tools helped rationalize observed degradation behavior and support impurity identification in molecules lacking comprehensive monographs.

### 2.2. cQA and CPP Selection for Stability-Justified Compounding

Elucidating degradation products and potential intermediates allowed us to assess them with in silico tools. Potential toxicological risks were flagged by cross-referencing with known structural alerts (e.g., nitroso, quinone, and aldehyde fragments) using in silico toxicology platforms such as Derek Nexus (Version 6.4.1) [33], TEST (version 5.1.2) [34], and Toxtree (version 3.1.0) [35]. These preliminary assessments informed which degradants warranted more stringent limits as part of cQAs, by using decision impurity management decision tree such as that proposed by Bercu et al. [36].

The identification of cQAs for degradation-related impurities based on toxicological relevance, physicochemical impact, and formation kinetics. To maintain these cQAs, we defined CPPs informed by the degradation studies. These included excipient selection, pH control, compounding sequence, container type, and maximum allowable storage time. In the absence of pharmacopoeial standards, this combined cQA-CPP strategy provided a Quality-by-Design (QbD) approach for validating hospital compounding protocols and ensuring consistent product quality under real-world conditions.

## 3. Results and Discussion

### 3.1. Outcomes from Our Prior Studies on Drug Degradation

The variability of degradation profiles observed across the tested compounds reinforced the necessity of a molecule-specific, risk-based formulation strategy. Table 1 summarizes the key degradation pathways, identified impurities, targeted cQAs, and risk mitigation strategies applied to each molecule based on our prior studies.

#### 3.1.1. Degradation Process Highlighted in the Studies

For instance, apixaban underwent rapid hydrolysis under acidic and alkaline aqueous conditions. Structural elucidation of the main degradation products by LC–MS/MS analysis revealed that hydrolysis of the oxopiperidine moiety of apixaban occurred in acidic medium, whereas that of the tetrahydro-oxo-pyridine moiety would further happen under alkaline conditions [37]. The literature corroborated apixaban’s acid sensitivity, particularly during solution preparation and extended contact with aqueous media [38].

Conversely, ruxolitinib showed pronounced degradation under ambient light [39]. Mass spectrometry data indicated hydroxylated metabolites likely arising from aromatic ring photooxidation [39]. These pathways necessitated strict oxygen and light protection. This finding aligns with prior industrial stability data indicating poor resilience of Janus kinase inhibitors with respect to light [40].

Photolability was also evident for molecules such as argatroban and ONC201. Argatroban displayed a significant absorbance from 300 nm to 350 nm and formed aromatic hydroxylation products under daylight simulation [41]. ONC201, a new anticancer drug of the imipridone class, exhibited rapid photodegradation [42].

#### 3.1.2. Role of Theoretical Chemistry in Degradation Pathway’s Characterization

The integration of theoretical chemistry with forced degradation experiments was instrumental in informing formulation and risk-mitigation strategies. Computational modeling, especially mapping of electronic densities of molecules, provided early identification of moieties liable to degradation by specific stress, such as electrophilic aromatic rings, hydrolysis-prone esters, and oxidation-sensitive heterocycles, which aligned with experimentally observed degradation products. This concordance supported incorporating ab initio tools as a pre-screening step in hospital compounding risk assessments, particularly in the absence of full regulatory monographs.

For instance, analysis of mapped electronic spin densities for ruxolitinib [39] revealed potential for electrophilic aromatic substitution and oxidation at the pyrrolopyrimidine scaffold. These alerts were consistent with hydroxylated degradants detected by LC-HRMS in photolytic stress studies [39].

In another study, the selective hydrolysis process of nirmatrelvir, involving only two out of its four hydrolyzable groups, was investigated by mapping electrostatic potentials and Fukui function [43]. The theoretical results correlated well with the observed outcomes of forced degradation studies [43].

#### 3.1.3. Risk Assessment of Degradation Implications

In silico tools such as Derek Nexus, Toxtree, and TEST provided insights into the mutagenic or reactive potential of degradation products [44]. These alerts prompted us to include analytical monitoring points for certain impurities, even at early formulation stages, to ensure alignment with ICH Q3A/B impurity qualification thresholds [45,46].

In our studies, structural elucidation of the degradation products paved the way for structure based toxicological assessment. For instance, in silico studies of ONC201 and ruxolitinib degradation products revealed structurally alerting moieties [39,42]. In another study, an aromatic hydroxylamine degradant of argatroban raised concerns because alkylanilines have been associated with DNA-damage–mediated carcinogenicity [47]. These flagged impurities were evaluated under the ICH M7 framework as potential mutagenic risks and were prioritized for monitoring and control. The formation of degradation products with potential mutagenicity in 4 out of 8 the studies where we carried out this assessment (Table 1) supported combining structural elucidation with in silico toxicology into the design of cQA profiles, especially when compounding is performed in the absence of standardized regulatory monographs.

#### 3.1.4. Summary of Results and Pharmaceutical Implications

Each observed degradation behavior triggered a tailored risk assessment and informed a compound-specific formulation strategy (Table 1) as a function of compounded formulation type. For apixaban, buffering to pH 3.5–4.5 was proposed to slow acid-catalyzed hydrolysis, with the use of plastic-free, sealed containers to reduce sorption and catalysis. Reconstitution of argatroban injectable formulation prepared in advance was modified to include nitrogen flushing and light protection via amber glassware. Ruxolitinib and etoposide required compounding under subdued light, followed by immediate sealing in light-resistant packaging. In all cases, recommendations were adapted not only to manufacturing but also to storage and administration strategies, ensuring continuity of risk mitigation through the clinical use chain.

**Figure 1:**
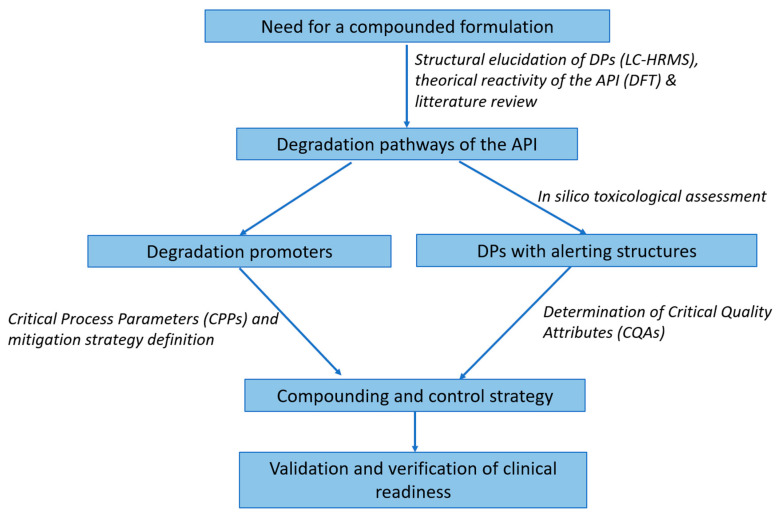
Risk-based formulation decision tree for hospital compounding. This stepwise model helps hospital pharmacists assess degradation liability (hydrolysis, oxidation, photolysis) based on experimental and in silico data, triggering tailored formulation controls. Decision nodes guide buffer selection, excipients, containers, protection measures, and shelf-life limits. Structural alerts (e.g., via Derek Nexus or Toxtree) prompt enhanced monitoring or toxicological review.

We consolidated these strategies (Table 1) into a visual risk-based decision tree (Figure 1, Section 3.2). Together, they provided an evidence-based, clinically actionable roadmap.

### 3.2. Risk-Based Scientific Framework for Formulating Personalized Medicines

#### 3.2.1. Proposed Risk-Based Decision Tree for Hospital Compounding

Building on Section 3.1, incorporating theoretical chemistry and in silico tools strengthens pharmacists’ ability to anticipate and control degradation-related risks. Beyond retrospective justification, these approaches pave the way for prospective formulation planning, allowing real-time risk mitigation aligned with ICH Q8–Q11 [23,24,25,26] principles. Moreover, by reducing dependence on trial-and-error laboratory testing, this hybrid predictive, empirical framework supports faster and safer deployment of personalized formulations in clinical settings.

For instance, in a pediatric emergency setting, if a new antiviral compound is required for extemporaneous preparation, computational profiling can flag degradation liabilities and guide safe compounding, even before full ICH stability data are available. This bridges the time gap between regulatory approval and bedside application, especially in institutions equipped with 3D printing technologies.

To operationalize the integration of degradation science, in silico predictions, and formulation control strategies, we propose a structured Risk-Based Decision Tree (Figure 1) designed for use by hospital pharmacists and pharmaceutical scientists. This tool translates degradation knowledge into actionable formulation and compounding decisions under real-world constraints.

***Inputs to the decision model***

The tool begins by consolidating the following data elements:

- Drug characteristics: chemical class, functional groups, known degradation pathways, and physicochemical properties (e.g., pKa, logP, solubility).
- Degradation data: experimental forced degradation profiles and/or predicted liabilities via theoretical modeling (Section 2.1).
- Compounding context: target patient group (e.g., pediatric, oncology), personalization level (dose flexibility, dosage form), shelf-life expectations, container availability, and administration route.

***Risk classification and triggering rules***

The input data are used to classify the compound under dominant degradation liabilities and potential issues related to degradation:

- Hydrolytic sensitivity (e.g., ester, amide cleavage)
- Oxidation-prone structures (e.g., phenols, heteroaromatics)
- Photolability (e.g., aromatic chromophores with absorbance above 290 nm)
- Structural alerts flagged by in silico software (e.g., Derek Nexus or Toxtree), interpreted per ICH M7(R2) for mutagenic potential

Each degradation class is matched with critical thresholds (e.g., impurity formation > 0.1% in 24 h, or DFT-predicted high reactivity) that trigger enhanced formulation controls.

***Decision outputs***

For each risk profile, formulation adjustments and compounding recommendations can be implemented:

- Formulation strategy: adjust pH (e.g., buffer apixaban to pH 3.5–4.5), use stabilizers (e.g., antioxidants for ruxolitinib), and avoid solvents promoting hydrolysis.
- Container and protection: amber glass for photolabile or oxidative drugs, nitrogen flushing for oxidizable APIs, and plastic-free vessels to avoid catalysis or adsorption.
- Workflow controls: light-protected or closed-system compounding, short beyond-use dating for unstable compounds, and on-demand 3D printing adaptation where suitable.

#### 3.2.2. Stability-Personalization Matrix for Oral Formulation Triage

To guide decision-making under resource constraints, a two-axis matrix can be applied (Table 2).

This matrix helps triage formulation complexity in settings with limited analytical capacity or turnaround requirements. By combining this decision matrix with the clinical need and the available facilities, the pharmaceutical dosage form to be implemented is determined pragmatically, as it is currently the case at Gustave Roussy hospital (Table 2).

#### 3.2.3. Use Case Illustration

***Apixaban***: high hydrolytic liability (Section 3.1.1; Table 1) → decision tree selects buffered SSE printable ink and short BUD (beyond-use date).

***Ruxolitinib***: Oxidation/photolysis risk with mutagenicity alerts (Section 3.1.1, Section 3.1.2, Section 3.1.3; Table 1) → nitrogen flush, antioxidant, light protection, and targeted stability testing.

These case-based outcomes show the clinical feasibility and regulatory alignment of the proposed tool.

## 4. General Discussion and Outlook

The demand for personalized medicine, particularly in hospitals, requires compounding frameworks that go beyond empirical preparation. They must be data-informed and risk-managed. While previous publications have characterized degradation pathways or proposed computational predictions in isolation, our framework is, to our knowledge, the first to integrate forced degradation data, ab initio modeling, and in silico toxicological alerts into a unified decision tool for hospital compounding (Figure 1). This holistic approach bridges the gap between industrial stability studies and the real-world constraints of personalized medicine at the bedside. This methodology may help to determine actions based on risk and personalization need (Table 2).

A central contribution of this work is its focus on degradation risk as a primary driver for formulation. These findings are not only of mechanistic interest but also directly translatable to daily hospital pharmacy practice. By systematically linking each degradation profile with actionable mitigation strategies (e.g., pH adjustment, nitrogen flushing, amber glass protection), our results demonstrate how laboratory insights can be operationalized into safe, reproducible compounding workflows. This practical dimension is often missing in purely analytical or computational studies.

Conventional pharmacopeial approaches assume validated shelf-life data and industrial-scale manufacturing. Hospital compounding often lacks both, especially for unlicensed pediatric uses, oncology-adjunct medicines, or off-label doses. The absence of monographs or complete stability data creates blind spots that increase the risk of impurity formation, chemical instability, and toxicity. By embedding degradation knowledge, such as apixaban hydrolysis or ruxolitinib oxidation, into formulation and container choices, the framework rationalizes clinical risk under tight timelines.

Computational approaches are more than predictive; they enable risk triage. When guided by ICH M7 and Q3A/B, they support impurity qualification and specification early in hospital formulation. This is valuable even when analytical resources are scarce. As in silico tools like Derek Nexus and TEST become more accessible, pharmacists can use them for both genotoxicity alerts and real-time risk flagging during formulation.

The decision tree (Section 3.2.1) is a practical translational step. It offers a structured process for selecting buffers, excipients, packaging, and shelf-life based on identified risks. By linking laboratory evidence with process control, the tool shortens decision time and improves reproducibility. In settings where resources are limited, it provides a clear, evidence-based pathway that supports safe, effective personalized medicines.

In our laboratory, retrospective application of the framework to hospital-compounded formulations identified all cases where early degradation led to shortened beyond-use dates (BUDs) or batch rejection. In a pilot with six newly compounded formulations, the framework’s risk assessment aligned well with observed outcomes under accelerated conditions (Table 3). Although these data are limited, they suggest that the approach can reduce stability-related failures and guide more robust formulation design. Larger, prospective studies are planned to quantify these benefits.

The concordance between predicted and observed degradation pathways across multiple molecules (Table 3) provides preliminary evidence of the robustness of the framework. Importantly, the methodology is not molecule-specific: it can be generalized to other APIs lacking pharmacopeial data, thereby offering hospital pharmacists a scalable strategy to anticipate and control chemical risks in compounded medicines.

## 5. Limitations and Considerations

While the proposed framework bridges a critical gap in hospital compounding by integrating degradation risk and in silico toxicology, several limitations should be acknowledged. First, toxicological qualification of degradation products, especially those flagged as potentially mutagenic (per ICH M7), may require in vitro or in vivo validation that is not feasible within the operational scope of most hospital pharmacies. In silico systems (e.g., TEST, Derek Nexus, Toxtree) are screening tools for mutagenicity, not definitive risk classifiers. A toxicology expert can provide a broader assessment across additional endpoints. As such, flagged impurities should be interpreted in context and, where necessary, escalated to institutional quality or pharmacovigilance bodies for further risk management.

Second, although the decision tree and scoring matrix are designed to support real-time formulation decisions, they currently depend on the availability of experimental degradation data or sufficient computational infrastructure. For APIs (active pharmaceutical ingredients) lacking published degradation profiles, the accuracy of recommendations may be reduced unless supported by other relevant inputs.

Lastly, the clinical application of 3D printing technologies, although promising, remains limited by formulation constraints, regulatory ambiguity, and material validation requirements, particularly when extending beyond the scope of investigational or early-phase studies.

While advanced quantum chemical calculations (e.g., DFT) and in silico toxicology tools can seem resource-intensive, scalable pathways exist for their adoption in hospital compounding. For high-complexity centers or academic hospital pharmacies, direct in-house use of open-source packages (e.g., ORCA [48] for DFT calculations, Avogadro [49] for structure building) is feasible with modest workstation hardware and pharmacist training. For medium and small hospitals, a tiered approach can be adopted: (1) access pre-compiled degradation liability datasets for common APIs, (2) use simplified cloud-based tools that require only structural input (e.g., TEST [34], SwissADME [50], Toxtree [35]), and (3) collaborate with regional or academic hubs for more detailed quantum calculations on-demand. This ensures that even facilities without advanced computational infrastructure can integrate structural alert screening and basic electronic descriptor analysis into their formulation risk assessment. Over time, shared repositories of calculated descriptors and degradation case studies can reduce the need for repeated calculations, making the process faster and more cost-effective.

## 6. Conclusions

This article presents a scientifically grounded and clinically actionable framework for risk-based formulation in hospital compounding, addressing a critical gap in the integration of degradation knowledge, toxicological alerts, and formulation design. Built on forced-degradation studies and in silico modeling, the framework supports proactive decisions that improve the safety, stability, and traceability of patient-specific medications, especially when pharmacopeial data are lacking.

By translating ICH principles (Q8–Q12, M7) into hospital-appropriate strategies, the framework enables the rational selection of formulation parameters such as pH, excipients, containers, and processing conditions. It introduces a structured approach to defining and controlling critical quality attributes (cQAs) and critical process parameters (CPPs) based on degradation risks and predicted toxicological liabilities. The integration of these elements with personalized manufacturing technologies, most notably semi-solid extrusion (SSE) 3D printing, extends the model into new domains of individualized therapy, including pediatric oncology and metabolic care.

Importantly, this approach shifts hospital compounding from a reactive practice to a forward-looking, data-informed formulation process. It empowers pharmacists and clinicians to deliver safer and more adaptive medications, with formulation strategies informed by mechanistic degradation insights and supported by modern predictive tools. As regulatory and clinical landscapes evolve toward precision and patient-centered care, the proposed framework stands as a valuable contribution to the future of decentralized pharmaceutical manufacturing.

Future work should focus on validating this model across diverse therapeutic areas, integrating real-world stability data with in silico predictions, and aligning outputs with regulatory expectations for hospital compounding, including decentralized manufacturing. The development of digital platforms embedding predictive toxicology, risk-scoring algorithms, and formulation databases could provide pharmacists with practical, user-friendly tools to implement the framework in daily compounding activities.

## Figures and Tables

**Table 1:** Summary of degradation risks and corresponding formulation, packaging, and administration adaptations applied to studied molecules, based on forced degradation studies and hospital compounding simulations.

API	Degradation Pathway	Key Degradants	Mutagenicity Alert?	Targeted CPPs	Mitigation Strategy
Apixaban	Acid/base-catalyzed hydrolysis	Open-ringed lactams	No	pH buffering; aqueous exposure time	Buffer to pH 3.5–4.5; use sealed containers; limit aqueous prep time
Argatroban	Photodegradation	Aromatic hydroxylamines	Yes	Light protection; BUD limitation	Prepare in subdued light; use within 24 h; store in amber syringes
Etoposide	Acidic hydrolysis + photolysis	Quinone species	No	Light avoidance; pH control	Prepare under low light; buffer pH; refrigerate
Nirmatrelvir	Acid and base cata-lysed hydrolysis	Hydrolyzed nitrile and/or trifluoroacetamide	No	pH control	Buffer pH to 3.0–5.0
ONC201	Oxidation + photolysis	Altered imidazole, tetrahydropyridine and piperidine rings	Yes	Light avoidance, antioxidant	Store in UV-blocking container; citric acid
Ruxolitinib	Oxidation + photolysis	Hydroxylated aromatics	Yes	Nitrogen flush; antioxidant; light protection	Flush with N_2_; include antioxidant; store in amber glass
Selumetinib	Photodegradation	Oxidized oxoamide	No	UV protection; fast administration	Store in UV-blocking blister; use within 24 h post-compounding
Umifenovir	Oxidation	Sulfoxide/sulfone	No	Headspace minimization; antioxidant buffer	Seal containers; use low-O_2_ buffers

**Table 2:** Decision matrix for balancing degradation risk and personalization need in hospital compounding. This table provides a four-quadrant framework to guide formulation strategy decisions based on the combined assessment of chemical stability risk and clinical personalization requirements. It enables hospital pharmacists to prioritize full profiling and mitigation efforts (e.g., advanced compounding, in silico evaluation, or 3D printing) in cases where both degradation sensitivity and personalization need are high, while streamlining efforts where risks or needs are minimal.

Stability Risk (Degradation Profile)	Personalization Need	Action	Type of Compounded Drug Product Implemented in Gustave Roussy Hospital
High (rapid degradation, genotoxic alerts)	High (pediatric, rare dose)	Full profiling, strict CPP control	3D printed formulations
High	Low	Minimal personalization, consider alternative therapies	If no adapted alternative exists, oral solid dosage forms (capsules or 3D printed formulations)
Low	High	Flexible format, but reduced need for full degradation profiling	Oral liquid formulation or 3D printed formulations
Low	Low	Standard compounding procedures acceptable	Oral liquid or solid oral dosage forms

**Table 3:** Preliminary validation of the proposed risk-based formulation framework using pilot prospective data from hospital-compounded medicines. Predicted degradation pathways were compared with observed degradation outcomes from stability testing under accelerated conditions. Correct predictions indicate full concordance between predicted and observed primary degradation mechanisms.

Formulation	Predicted Risk Pathway	Observed Degradation	Outcome
Apixaban suspension	Hydrolysis	Hydrolysis	Correct prediction
Ruxolitinib solution	Oxidation and photolysis	Oxidation and photolysis	Correct prediction
Argatroban infusion	Hydroxylamine formation	Hydroxylamine formation	Correct prediction
Nirmatrelvir oral liquid	Acid/base-catalyzed hydrolysis	Hydrolysis	Correct prediction
Custom pediatric amlodipine	Photodegradation	Photodegradation	Correct prediction
Voriconazole SSE printable ink	Oxidation	No significant degradation	No degradation observed (framework flagged as possible risk)

## Data Availability

Dataset available on request from the authors.
